# Nanopore sequencing of single-cell transcriptomes with scCOLOR-seq

**DOI:** 10.1038/s41587-021-00965-w

**Published:** 2021-07-01

**Authors:** Martin Philpott, Jonathan Watson, Anjan Thakurta, Tom Brown, Tom Brown, Udo Oppermann, Adam P. Cribbs

**Affiliations:** 1 Botnar Research Centre, Nuffield Department of Orthopedics, Rheumatology and Musculoskeletal Sciences, National Institute of Health Research Oxford Biomedical Research Unit (BRU), University of Oxford, Oxford, UK; 2 Oxford Centre for Translational Myeloma Research University of Oxford, Oxford, UK; 3 ATDBio, Oxford, UK; 4 Radcliffe Department of Medicine, Oxford University, Oxford, UK; 5 Translational Medicine, Bristol Myers Squibb, Summit, NJ USA; 6 Chemistry Research Laboratory, Department of Chemistry, University of Oxford, Oxford, UK; 7 Centre for Medicines Discovery, University of Oxford, Oxford, UK

**Keywords:** Transcriptomics, RNA sequencing

## Abstract

Here we describe single-cell corrected long-read sequencing (scCOLOR-seq), which enables error correction of barcode and unique molecular identifier oligonucleotide sequences and permits standalone cDNA nanopore sequencing of single cells. Barcodes and unique molecular identifiers are synthesized using dimeric nucleotide building blocks that allow error detection. We illustrate the use of the method for evaluating barcode assignment accuracy, differential isoform usage in myeloma cell lines, and fusion transcript detection in a sarcoma cell line.

## Abstract (web summary)

Single-cell transcriptomes are accurately sequenced with error-tolerant barcodes.

## Main

**Fig. 1:**
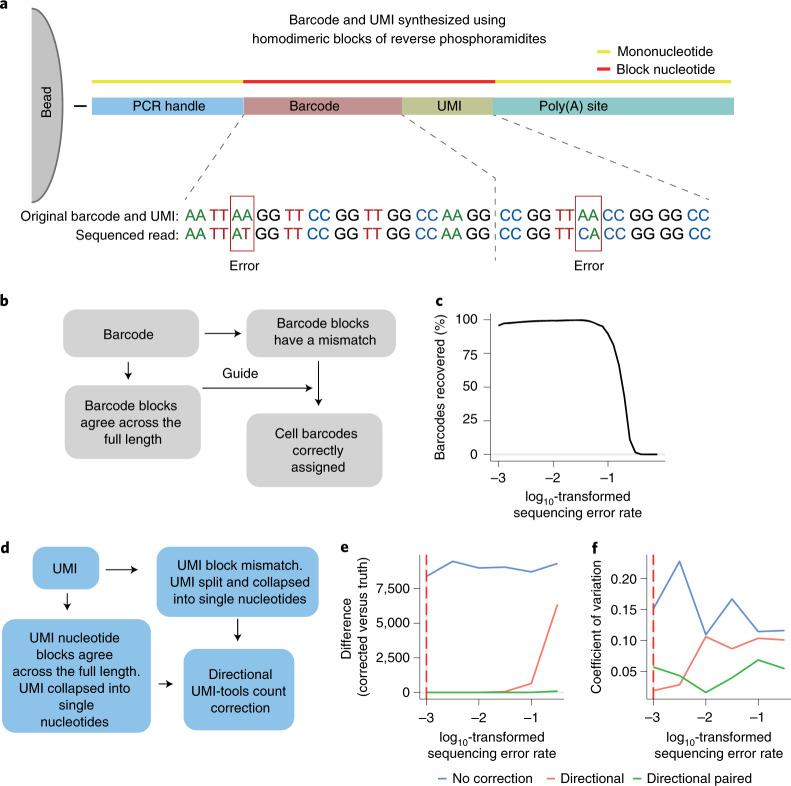
Developing a strategy to error-correct barcode and UMI sequences from droplet-based sequencing. **a**, Schematic bead and oligonucleotide structure using dimer blocks of nucleotides for Buc-seq. **b**, Cell barcode-assignment strategy. **c**, UMI deduplication strategy. **d**, Simulated data showing the number of barcodes recovered with increasing simulated sequencing error rates. **e**,**f**, Simulated data showing the difference and coefficient of variation between the deduplicated UMIs and the ground truth. Correction of the UMI counts was performed using a basic directional network-based approach after accounting for sequencing errors within homodimeric blocks of nucleotides.

Long-read sequencing technologies such as PacBio single-molecule real-time (SMRT) sequencing^1^ or Oxford Nanopore sequencing^2^ enable the sequencing of full-length transcripts. The application of PacBio SMRT to single-cell sequencing has been hindered by a low sequencing capacity (four million reads per flow cell), which means that a single run can report on only 40–133 cells at a comparable read depth to short-read approaches, or on thousands of cells by sacrificing quantitative information^3^. Zeng et al.^4^ have improved the platform for single-cell sequencing workflows by concatenating multiple full-length cDNAs into a single insert. This approach led to a return of 10 million reads from a single SMRT cell, providing eight times more data output than the standard PacBio sequencing protocol. Alternatively, nanopore sequencing provides up to 250 million reads per PromethION flow cell, but its main drawback is its high error rate compared with both PacBio long-read and Illumina short-read sequencing (5–15% compared with less than 1%)^5^. To overcome the low base-calling accuracy of Oxford Nanopore sequencing, here we describe single-cell corrected long-read sequencing (scCOLOR-seq), in which the barcode and unique molecular identifier (UMI) regions of the oligonucleotide-barcoded RNA-capture microbeads are synthesized using homodimeric nucleoside phosphoramidite building blocks (Supplementary Fig. 1), which provides a means for sequencing-error detection and correction of the barcode and UMI (Fig. 1a).

To correctly assign barcodes to cells, a computational strategy was developed in which barcodes were identified in a two-pass assignment method (Fig. 1b and Supplementary Fig. 2). First, barcodes without errors were identified on the basis of nucleotide pair complementarity across the full length of the barcode. Next, these accurate barcodes were used as a guide to correct the remaining erroneous barcodes. Using simulated data, we show that this strategy is capable of correcting erroneous barcodes with a high sequencing error rate, with 96% of barcodes recovered with a barcode sequencing error rate of up to 10% (Fig. 1c). In single-cell sequencing, UMIs allow the deduplication of single transcripts that are detected multiple times in sequencing, arising from PCR copies produced during library preparation. The directional network-based method first proposed by UMI-tools^6^ was modified to correct for UMI sequence duplication (Fig. 1d and Supplementary Fig. 3). Using simulated data, we show that the dimer-synthesized UMIs can be used to deduplicate UMIs even with a sequencing error rate above 10% (Fig. 1e,f and Supplementary Fig. 4).

scCOLOR-seq was validated using human HEK293T and mouse 3T3 single-cell Drop-seq libraries from approximately 1,200 cells (at a 50:50 mouse:human cell ratio), followed by Illumina short-read sequencing. Overall, 68% of all reads show complete dinucleotide block complementarity across the full barcode sequence. This suggests that the theoretical base-calling accuracy for the barcode should be 98.4%, which aligns with the reported accuracy of Illumina sequencing. The dimer-correction approach was evaluated by measuring the proportion of human, mouse and mixed species cells identified following increased edit distances (that is, the Levenshtein distance) between the error-sequenced and the accurately sequenced barcodes (Supplementary Fig. 5). An edit distance of 4 was found to result in accurate assignment of both mouse and human reads (Supplementary Fig. 6), enabling the recovery of an extra 8% of total reads. Although further reads could be recovered using an edit distance of 5, this was obtained at the expense of increased numbers of mixed species cells (Supplementary Fig. 5i).

**Fig. 2:**
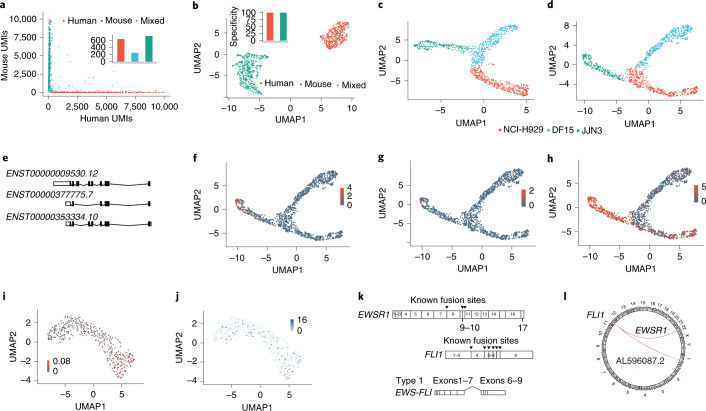
scCOLOR-seq identifies transcript isoform diversity and fusion transcripts in cancer cell line models. **a**,**b**, Human HEK293T and mouse 3T3 cells were mixed at a 1:1 ratio and approximately 1,200 cells were taken for encapsulation and cDNA synthesis followed by nanopore sequencing. **a**, A Barnyard plot showing the expression of mouse and human UMIs before quality filtering using an edit distance of 6. **b**, A UMAP plot of data after quality filtering showing the clustering of human, mouse or mixed human and mouse cells after barcode correction using an edit distance of 6. Insets: bar plots show the specificity of UMIs aligning to either the human or mouse UMAP cluster. **c**–**h**, NCI-H929, DF15 and JJN3 myeloma cell lines were mixed at a 1:1:1 ratio and approximately 1,200 cells were taken for cDNA synthesis and sequenced using a PromethION flow cell. **c**,**d**, UMAP plot of gene expression (**c**) and transcript isoform expression (**d**). **e**, Principal *CD74* (also known as *HLA-DR*) splice variants showing all protein-coding transcripts. **f**–**h**, UMAP plot showing the isoform expression of detected *CD74* transcripts *ENST00000377775.7* (**f**), *ENST00000353334.10* (**g**) and *ENST00000009530.12* (**h**). **i**, A UMAP plot of total fusion transcripts in Ewing’s cells mapped as a parentage of the total RNA of the cell. **j**, A UMAP plot showing the expression of the *EWS-FLI* fusion transcript. **k**, A schematic showing the structure of the *EWSR1* and *FLI1* genes. The *EWS-FLI* fusion transcript consists of the 5′ end of the *EWSR1* gene and the 3′ end of the *FLI1* gene. Arrowheads denote known fusion events and the most common type-1 fusion transcript is shown. **l**, A circular representation of the fusion transcripts identified between *FLI1* and *EWSR1*.

Application of scCOLOR-seq to nanopore sequencing identified the presence of a poly(A) sequence in 40% (range, 24–62%) of all nanopore sequencing reads and detected 12.9% (range, 9–15%) of these reads with dual nucleotide complementarity across the full barcode sequence (Supplementary Fig. 7). This suggests that the theoretical base-calling accuracy of single-cell nanopore sequencing should be 91.8%. However, barcodes were observed to often contain more than one error per barcode, which has the effect of reducing the overall measurable base-calling accuracy to 86% (Supplementary Fig. 8). Naive collapsing of barcodes and UMIs sequenced into single-base sequences without error correction led to only 81 recovered cells (Supplementary Fig. 9). The dimer correction approach using an edit distance of 6 led to the recovery of 54% (range, 43%–68%) of barcodes containing sequencing errors (Fig. 2a,b and Supplementary Fig. 7c). Increasing the edit distance to 7 increased recovery to 82% (range, 79.8%–83.6%), at the expense of slightly increased numbers of mixed cells (Supplementary Fig. 10). However, filtering on the basis of at least the presence of 200 features per cell removed a substantial proportion of mixed cells (Supplementary Fig. 11). Cells were then projected into two dimensions using uniform manifold approximation and projection (UMAP) and a clear separation of the mouse and human cell populations was observed, with 1,077 cells recovered using an edit distance of 6 and 1,064 cells recovered using an edit distance of 7 (Supplementary Fig. 10).

scCOLOR-seq was applied to a mixture (1:1:1 ratio) of human NCI-H929, JJN3 and DF15 myeloma cell lines and approximately 500 cells were sequenced using a MinION flow cell and 1,200 cells sequenced using a PromethION flow cell. After filtering with a minimum of 200 features per cell (Supplementary Figs. 12, 13), we show that nanopore sequencing can resolve the different myeloma cell types at both the gene level (Fig. 2c and Supplementary Fig. 14) and the transcript level (Fig. 2d and Supplementary Fig. 14b–f). There was also a good correlation between Oxford Nanopore and Illumina gene counts per cell (*R* = 0.67) and the number of UMIs per cell (*R* = 0.65) (Supplementary Fig. 15d,e). The clustering is more defined at the transcript level and more diffuse at the gene expression level, likely reflecting the diversity of transcript use within these cells. We next searched for differentially regulated transcripts between cell types and clusters. In this experiment, cell-type-specific usage was observed for 359 genes and 416 differentially expressed isoforms. Differential transcript usage was particularly apparent for the marker *CD74* (Fig. 2e–h), which is a potential therapeutic target in multiple myeloma^7^. Furthermore, in agreement with the literature and the biology of plasma cells^8^, a differential expression of both immunoglobulin κ and λ light-chain isoform use between the different myeloma cell lines was observed (Supplementary Figs. 16, 17).

Long read sequencing permits the measurement of fusion transcripts that are often key drivers of tumor development. To illustrate the principle, Ewing’s sarcoma was selected, which harbors the t(11:22)(q24:q212) translocation that generates *EWS-FLI*, a fusion between *EWSR1* (Ewing’s sarcoma breakpoint region 1) and the ETS transcription factor *FLI1* (Friend leukemia integration 1) genes^9^. The EWS–FLI protein regulates the expression of numerous target genes that promote cancer survival and drug resistance^10,11^. scCOLOR-seq was performed on human STA-ET-1 Ewing’s cells, which are known to express the EWS–FLI protein and we measured the presence of the *EWS-FLI* fusion transcript within each single cell. Given that fusion transcripts can be falsely detected as a consequence of PCR artifacts^12^, the mixed-species data (Fig. 2a,b) was first used to determine the frequency of false-positive fusion events. As these would never occur naturally, the incidence of human to mouse fusion events can be used as a guide to set filtering thresholds. The mixed species data reveals that 5% of total reads contain a fusion event, with 35% of these reads showing mixed human and mouse fusion transcripts (Supplementary Fig. 18). This suggests that over 70% of detected fusion transcripts could be PCR artifacts. However, application of a filtering threshold based on a minimum of 5 UMIs for each fusion event removed all the mixed human–mouse fusion reads (Supplementary Fig. 18b,c), which was subsequently applied to eliminate false-positive fusion events in the Ewing’s cell data. After filtering, a total of 10,258 unique fusion transcripts were detected, enabling the measurement of the *EWS-FLI* fusion transcript in 17% of cells (Fig. 2i,j). Several *EWS-FLI* fusion transcripts have been reported^13^. We detected the presence of the most common type ‘1’ form in our single-cell data, consisting of the first seven exons of *EWSR1* joined to exons 6–9 of the *FLI1* gene (Fig. 2k and Supplementary Fig. 19). In addition, a potentially previously undescribed fusion transcript between *FLI1* and the long noncoding RNA AL596087.2 was observed (Fig. 2l).

Several groups have reported the use of short-read Illumina sequencing data to error-correct long-read Oxford Nanopore single-cell sequencing^3,14–16^, in which the more accurate barcode sequences from Illumina sequencing are used as a guide to assign Oxford Nanopore reads to cells. Although this approach was able to increase assignment rates from around 6% to more than 60%, the requirement to independently construct and sequence two libraries increases the cost of single-cell sequencing. Moreover, accurate UMI assignment is challenging with this approach because of the random nature of the UMI generation and the low base-calling accuracy of nanopore sequencing. Volden et al. used a rolling circle amplification to concatemeric consensus (R2C2) method to error-correct nanopore sequencing^17^. Although this method achieved 96% sequencing accuracy, this still only translated to 72% of barcodes demultiplexing correctly, with 45% of UMIs not matching against parallel Illumina sequencing. Furthermore, the increased read length that is needed to support this error-correction approach is prone to increased error rates for longer reads in the late stages of a sequencing run without reagent refueling^18^.

scCOLOR-seq has multiple advantages over current methodologies to correct error-prone sequencing. The method provides superior error correction of single-cell sequencing barcodes, with over 80% recovery of reads when using an edit distance of 7, or over 60% recovery when using a conservative edit distance of 6. Uniquely, UMIs can be deduplicated with a high level of accuracy, approaching 100% in simulated data. Furthermore, we envision that the method can be further improved using blocks of trimer phosphoramidites. However, at present, these are not commercially available and the synthesis of reverse trimer phosphoramidite blocks is considerably more complex than for dimers.

In summary, single-cell long-read technology has the potential to open new avenues within genomics. For example, we demonstrate that it is possible to measure fusion events in chimeric reads at the single-cell level, which is only practical with long-read technology. scCOLOR-seq provides a simplified and more robust method to perform quantitative long-read transcript sequencing on large numbers of cells. We propose the use of this approach to stimulate further work on single-cell copy number variation and mutational analysis, which would have considerable potential in diagnostics and for the understanding of human disease.

## Methods

### Cell lines and reagents

HEK293T, JJN3, H929 and 3T3 cells were purchased from the ATCC. DF15 cells were a gift from Celgene (now Bristol Myers Squibb). Cell lines were cultured in DMEM low-glucose medium supplemented with FBS for no more than 20 passages. The cells were mycoplasma tested routinely and authenticated by STR during the course of this project.

### Oligonucleotide synthesis

Solid-phase phosphoramidite oligonucleotide synthesis on Toyopearl HW-65S resin (Tosoh Biosciences, 0019815) was performed by ATDBio, in the 5′–3′ direction (using reverse amidites), using a method adapted from Macosko et al.^19^. The sequence of the capture oligonucleotide is as follows: Bead-5′-TT-[spacer]-TTTTTTTAAGCAGTGGTATCAACGCAGAGTACJJJJJJJJJJJJNNNNNNNNTTTTTTTTTTTTTTTTTTTTTTTTTTTTTT-3′, where ‘J’ indicates a dual nucleotide dimer block added via split and pool synthesis using reverse dimer phosphoramidites (Supplementary Fig. 1; purchased from ChemGenes as custom products), ‘N’ indicates a degenerate dimer nucleotide (added using an equimolar mixture of the four reverse dimer phosphoramidites,), [spacer] is hexaethylene glycol, added using DMT-protected hexaethylene glycol phosphoramidite (LGC Link, 2129), and the other bases are standard (monomeric) DNA bases, added using reverse amidites (LGC Link, 2022, 2021, 2023 and 2020). AAGCAGTGGTATCAACGCAGAGTAC is the PCR handle.

Before oligonucleotide synthesis, the initial loading of hydroxyl groups on the resin was reduced via a capping reaction. Capping was performed by suspending the resin in a 1:1 mixture of Cap A (tetrahydrofuran:lutidine:acetic anhydride 8:1:1) and Cap B (tetrahydrofuran:pyridine:1-methylimidazole 8:1:1) at room temperature for 24 h. After capping, oligonucleotide synthesis was performed using an ABI 394 DNA synthesizer, using a modified 1 μmol synthesis cycle (with an extended coupling time of 5 min for standard monomer bases and 10 min for dimer bases, spacers and linkers). The barcode was generated using 12 split-and-pool synthesis cycles. Before the first split-and-pool synthesis cycle, beads were removed from the synthesis column, pooled and mixed, and divided into four equal aliquots. The bead aliquots were then transferred to separate synthesis columns before coupling with the dimer reverse amidite. This process was repeated 11 times. Following the final split and pool cycle, the resin was pooled, mixed and divided between four columns, ready for the next part of the synthesis. An equimolar mixture of the four dimer phosphoramidites was used in the synthesis of the degenerate UMI (poly(N)) region, and (monomeric) T reverse amidite was used for the poly(T) tail. After oligonucleotide synthesis, the resin was washed with acetonitrile and dried before deprotection in aqueous ammonia (55 °C, 6 h).

### Simulated barcode data

Barcode sequences were simulated with a length of 24 (12 blocks of nucleotides pairs) and then imitated the process of randomly introducing PCR errors and sequencing errors into 95% of the barcodes. A two-pass barcode assignment strategy was then performed in which true barcodes were identified on the basis of the nucleotide pair complementarity across the full length of the barcode. These true barcodes were then used as a guide to correct the remaining barcodes on the basis of approximate string matching. String matching was performed using the Levenshtein edit distance, which is a metric for measuring the difference between two strings. The following values were used as values within the simulations: sequencing depth, 400; number of UMIs, 10–100; barcode length, 24; PCR error rate, 1 × 10^−5^; sequencing error rate, 1 × 10^−1^ to 1 × 10^−7^; and number of PCR cycles, 25.

### Simulated UMI data

Simulated UMI data were generated with a length of 16 (8 blocks of nucleotide pairs) to confirm the accuracy of the UMI correction method by mimicking UMI PCR amplification and sequencing errors seen with Oxford Nanopore sequencing. UMIs were generated following an approach that was initially proposed by UMI-tools^6^. In brief, each UMI was generated at random, with a uniform probability of amplification (0.8–1.0). PCR cycles were simulated so that each UMI was selected in turn and duplicated according to the probability of amplification. PCR errors were added randomly and then any new UMI sequences were assigned new probabilities of amplification. A defined number of UMIs were randomly sampled to simulate sequencing depth and sequencing errors introduced with a specified probability. Finally, the presence of mismatched double nucleotides within the UMI were checked for and if errors were detected, the UMIs were split into two and then separately collapsed into 8 bp nucleotides. Unambiguous UMIs were collapsed into 8 bp nucleotides without splitting. The number of true UMIs was then estimated from the final pool of UMIs using UMI correction methods proposed in the original UMI-tools manuscript^6^. The following values were used as values within the simulations. Sequencing depth, 10–400; number of UMIs, 10–100; UMI length, 6–16; PCR error rate, 1 × 10^−3^ to 1 × 10^−5^; sequencing error rate, 1 × 10^−1^ to 1 × 10^−7^; and number of PCR cycles, 4–12.

### Droplet-based single-cell RNA sequencing

Single-cell capture and reverse transcription were performed using the Drop-seq approach, as described previously^19^. In brief, cells were loaded into the DolomiteBio Nadia system microfluidic cartridge at a concentration of 310 cells per μl^20^. Oligonucleotide beads were synthesized by ATDBio. Beads were loaded into the microfluidic cartridge at a concentration of 620,000 beads per ml. Cell capture and lysis were performed according to the manufacturer’s instructions of the Nadia instrument (DolomiteBio). The droplet emulsion was then disrupted using 1 ml of 1*H*,1*H*,2*H*,2*H*-perfluoro-1-octanol (Sigma) and beads were released into aqueous solution. After several washes, the beads were subjected to reverse transcription. Before PCR amplification, beads were washed and then treated with ExoI exonuclease for 45 min. PCR was then performed using the SMART PCR primer (AAGCAGTGGTATCAACGCAGAGT) and cDNA was subsequently purified using AMPure beads (Beckman Coulter). To achieve a high concentration of cDNA the input was subjected to 25 cycles of PCR amplification, rather than the 13 stated in the original Drop-seq protocol. Finally, cDNA was quantified using a TapeStation (Agilent Technologies) using a DNA high-sensitivity D5000 tape before being split for Illumina or Oxford Nanopore library generation.

### Single-cell Illumina library preparation for sequencing

Library preparation for Illumina sequencing was performed as described previously^19^. In brief, purified cDNA was used as an input for the Nextera XT DNA library preparation kit (Illumina). Library quality and size were determined using a TapeStation (Agilent Technologies) high-sensitivity D1000 tape. High-quality samples were then sequenced to a minimum of 50,000 reads per cell on a NextSeq 500 sequencer (Illumina) using a 75-cycle High Output kit using a custom read-1 primer (GCCTGTCCGCGGAAGCAGTGGTATCAACGCAGAGTAC). The sequencing depth was around 50,000 reads per cell barcode.

### Nanopore library preparation for sequencing

Full-length cDNA samples were prepared using the Oxford Nanopore Technologies SQK-LSK-109 Ligation Sequencing Kit, with the following modifications. Incubation times for end preparation were increased to 15 min and all washes were performed with 1.8× AMPure beads to improve the recovery of smaller fragments. Short fragment buffer was used for the final wash of libraries. Next, 50 fmol samples of the library were sequenced on either a MinION FLO-MIN106D R9.4.1 flow cell or PromethION FLO-PRO002 R9.4.1 flow cell, according to the manufacturer’s protocol. A sequencing depth of 40,000 reads per cell was aimed for, which for 500 cells equates to two or three flow cells of a MinION (the final sequencing depth was at least 20 million). For 1,200 cells, sequencing was carried out using a PromethION flow cell across one flow cell (final read depth was at least 48 million).

### Illumina-based single-cell RNA-sequencing analysis workflow

The FASTQ data were processed using a custom-written cgatcore pipeline (https://github.com/Acribbs/TallyNN)^21^. Ambiguous and unambiguous reads were identified on the basis of the occurrence of dual nucleotide complementarity within the barcode sequence. The unambiguous barcodes were then used to error-correct the ambiguous reads by fuzzy searching using a Levenshtein distance of 4 (unless stated otherwise in the figure legend). The barcode and UMI sequence for the corrected read pairs were then collapsed into single-nucleotide sequences. The resulting FASTQ files were used as an input for Kallisto (v.0.46.1) bustools (v.0.39.3)^22^, which was used to generate a counts matrix. This counts matrix was used as an input for the standard Seurat pipeline (v.3.1.4)^23^.

### Nanopore-based single-cell RNA-sequencing analysis workflow

Base calling was performed on the FAST5 data to generate FASTQ files using Guppy (v4.2.2) (guppy_basecaller --compress-fastq -c dna_r9.4.1_450bps_hac.cfg -x “cuda:1”) in GPU mode from Oxford Nanopore Technologies running on a GTX 1080 Ti graphics card. After base calling and the generation of FASTQ files, for each read the barcode and UMI sequence were identified by searching for the poly(A) region and flanking regions before and after the barcode and UMI. Accurately sequenced barcodes were identified based on their dual nucleotide complementarity. Unambiguous barcodes were then used as a guide to error-correct the ambiguous barcodes in a second-pass correction analysis approach (Supplementary Fig. 2). Fuzzy searching was performed using an edit distance (a string matching algorithm for measuring the distance between two strings) of 6 (unless stated otherwise in the figure legend) and replaced the original ambiguous barcode with the unambiguous sequence. A white list of barcodes was then generated using UMI-tools whitelist (umi_tools whitelist --bc-pattern=CCCCCCCCCCCCCCCCCCCCCCCCNNNNNNNNNNNNNNNN --set-cell-number=1000)^6^. This white list was used to assess the ratio of the quality of cells to the read count and used as an input for UMI-tools extract. Next, the barcode and UMI sequence of each read were extracted and placed within the read2 header file using UMI-tools extract (umi_tools extract --bc-pattern=CCCCCCCCCCCCCCCCCCCCCCCCNNNNNNNNNNNNNNNN --whitelist=whitelist.txt). Reads were then aligned to the transcriptome using minimap2 (ref. ^24^) (-ax splice -uf --MD --sam-hit-only --junc-bed) using the reference transcriptome for human hg38 and mouse mm10. The resulting SAM file was converted to a BAM file and then sorted and indexed using samtools^25^. The transcript name was then added as a XT tag within the BAM file using pysam. Finally, UMI-tools count (umi_tools count --per-gene --gene-tag = XT --per-cell --double-barcode), with modifications that allow the program to handle oligonucleotide blocks, was used to count features to cells before being converted to a market matrix format. UMI-tools count was modified to handle the double nucleotide UMIs as defined below. This counts matrix was then used as an input into the standard Seurat pipeline.

### UMI error correction

UMI-tools was forked on GitHub (https://github.com/Acribbs/UMI-tools) and the counts functionality was modified to handle the double oligonucleotide design. In brief, if a UMI contained at least one sequencing error, the UMI was split into two and then separately collapsed into 8 bp nucleotides (Supplementary Fig. 3). UMIs that did not contain a sequencing error were collapsed into 8 bp nucleotides without splitting. The directional method implemented within the original UMI-tools was then performed to correct UMI sequencing errors.

### Dimensionality reduction and clustering

Gene and transcript expression matrices were generated by UMI-tools count (for Oxford Nanopore data) or kallisto bustools (for Illumina data) and were processed using R/Bioconductor (v.4.0.3) and the Seurat package (v.3.1.4). Cells that expressed fewer than 200 features were removed from the analysis and gene matrices were cell-level-scaled and log-transformed as per the standard Seurat workflow^23^. The top 2,000 highly variable transcripts or genes were selected using Seurat FindVariableFeatures function. Principal component analysis dimensionality reduction was then performed to identify features that contributed to sources of variation within the data. Clustering was performed within Seurat using the Louvain algorithm, an unsupervised hierarchical clustering algorithm implemented by default in the standard Seurat workflow. To visualize the single-cell data, data were projected onto a UMAP, which is a non-linear dimensional reduction technique^26^. Cell-type determination was performed using clustifyr v.1.0.0 to identify correlated gene expression between single cells and bulk RNA-sequencing gene lists from the harmonize database^27,28^.

### Differential gene and isoform expression

Differential expression analysis was performed using nonparametric Wilcoxon test on log_2_(transcript per million) expression values. Differentially expressed genes and transcripts were selected on the basis of an absolute log_2_-transformed fold change of >1 and an adjusted *P* value of *P* < 0.05.

### Identification of fusion transcripts

Nanopore reads were aligned to the hg38 genome with minimap2 (-map-ont --MD --sam-hit-only -junc-bed --secondary=no). The splice junction BED file was generated from the Gencode v.36 GTF file using paftools, the minimap2 companion software. The SAM file was filtered using samtools to remove all non-primary alignment and supplementary alignments (samtools view -F 3328). Chimeric reads were identified on the basis of the SA SAM tag, which lists all other supplementary alignments. All SAM file processing was performed using pysam v.0.15.2. Next, the SA tag was inspected and assigned to the genomic feature using a BED file containing records of all known coding genes. The SAM record was updated with Ta, Tb, Tc and Td tags, which define the gene positional information from the BED file. Finally, fusion transcripts were annotated with gene information and the barcode information was used to generate per-cell counts for each translocated read. The counts table was then merged with the original transcript Seurat object. Original UMAP embeddings that were calculated for the transcript-only level analysis were used for visualization.

PCR and nanopore sequencing artifacts must be taken into consideration when investigating previously undescribed isoforms or translocations. Most PCR duplications and artifacts can be eliminated when the UMI is accounted for, but some artifacts may remain. Identification of reverse-transcription artifacts are more difficult to identify because reverse transcription introduces template switching between homologous sequences leading to increased chimeric cDNA^12^. However, to minimize the false-positive translocations in the data, a thermostable reverse-transcription enzyme was used, exonic chimeric transcripts were removed and a minimum of 5 UMIs per translocation event was required.

### Base-calling accuracy

To calculate the theoretical base-calling accuracy from the frequency of reads that show perfect barcode dimer complementarity, we use the following equation: ${\text{Base-calling}}\,{\rm{accuracy}} = \root {l} \of {b}$, where *b* is the frequency of barcode complementarity and *l* is the length of the barcode sequence. To measure the barcode base-calling accuracy, each base pair was assessed for complementarity across the whole length of the barcode for every read in the FASTQ file. Accuracy was determined by counting the occurrence of errors and dividing this by the number of total bases within the total barcodes. This code is provided as a Jupyter Notebook within the TallyNN GitHub repository.

### Reporting Summary

Further information on research design is available in the Nature Research Reporting Summary linked to this article.

## Online content

Any methods, additional references, Nature Research reporting summaries, source data, extended data, supplementary information, acknowledgements, peer review information; details of author contributions and competing interests; and statements of data and code availability are available at 10.1038/s41587-021-00965-w.

## Supplementary information

Supplementary Information Supplementary Figs. 1–19.

Reporting Summary

## Data Availability

Sequencing data have been deposited in the GEO under accession number GSE162053.

## Code availability

All custom pipelines used for analyses are available on GitHub (https://github.com/Acribbs/TallyNN). Modifications to the UMI-tools code are also available as a fork on GitHub (https://github.com/Acribbs/UMI-tools).
