# Supplementary material for: Nanopore sequencing of single-cell transcriptomes with scCOLOR-seq
Source: Nat Biotechnol. 2021 Jul 1;39(12):1517–20. doi: 10.1038/s41587-021-00965-w (PMC8668430; doi:10.1038/s41587-021-00965-w)
Supplement: Supplementary file 1 — Supplementary Figs. 1–19. [file 41587_2021_965_MOESM1_ESM.pdf]

nature portfolio

https://doi.org/10.1038/s41587-021-00965-w

Supplementary information

Nanopore sequencing of single-cell transcriptomes with scCOLOR-seq

In the format provided by the authors and unedited

Supplementary Figures

Figure 1:

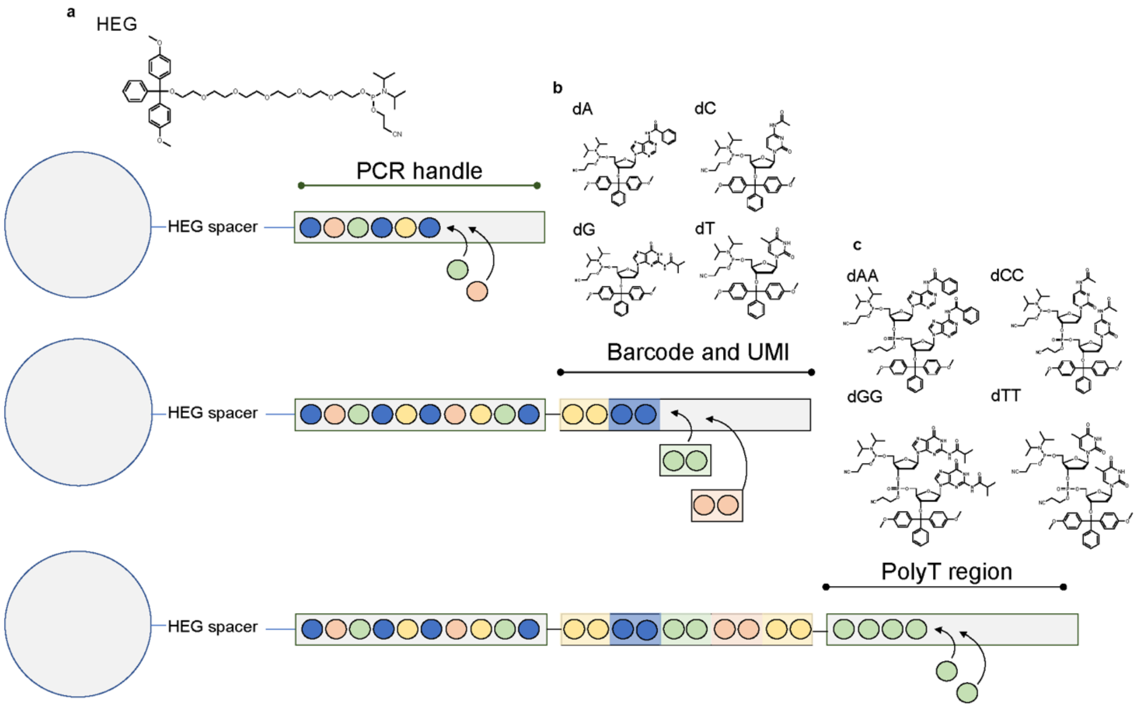

Barcode and UMI synthesis strategy

Solid-phase phosphoramidite oligonucleotide synthesis is performed on Toyopearl HW-65S resin. Following the attachment of a hexaethylene glycol (HEG) linker (**a)**, the PCR handle is synthesised using single reverse phosphoramidites (**b**). The barcode and UMI region of the capture oligonucleotide are formed of blocks of homodimer nucleotides (**c**), added using reverse dimer phosphoramidites. Finally, polyT oligonucleotide region is synthesised using single reverse phosphoramidites.

Figure 2:

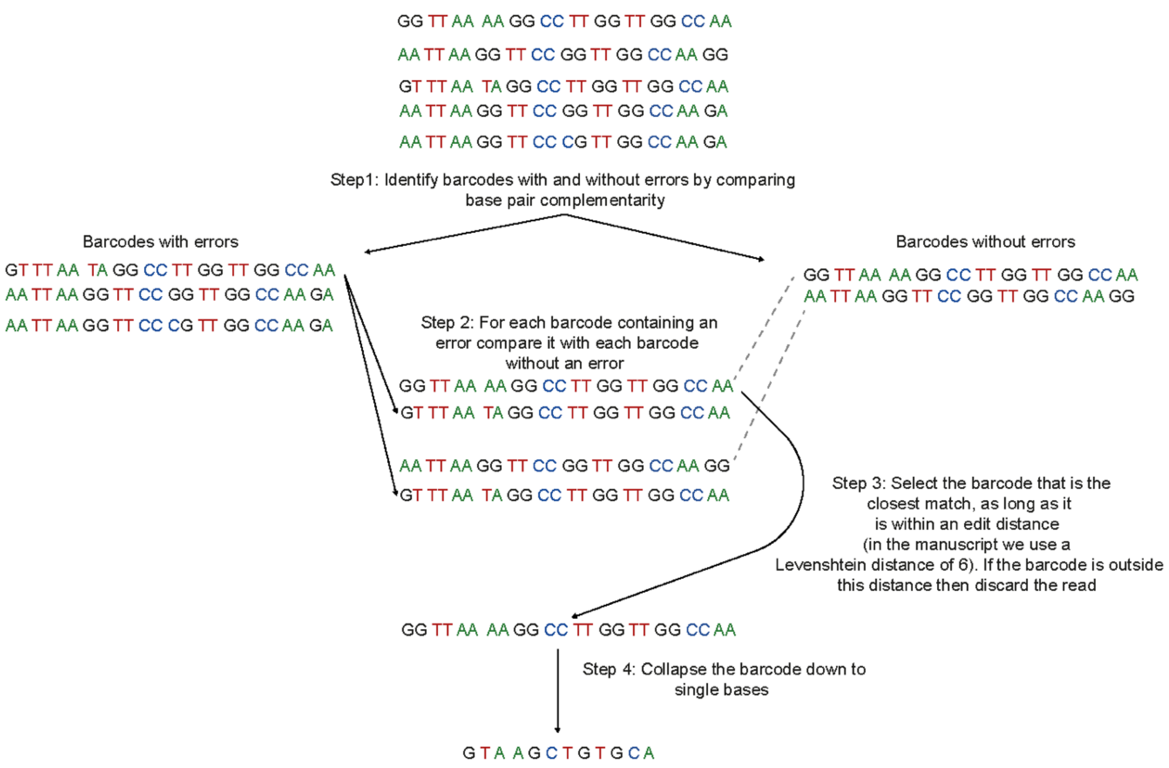

Barcode assignment strategy

Barcodes are assigned to cells by grouping reads that differ by no more than a specified edit distance (we typically use a Levenshtein distance of 6). Indels are typically not considered. The first step involves a first pass whitelisting approach where barcodes without errors are identified based on full base pair complementarity. Blacklisted barcodes containing errors are then compared to the whitelist of error free barcodes. Barcodes with the closest match to a barcode without an error are then selected, as long as it is within the specified edit distance. If the barcode is outside the specified edit distance, then the read is discarded.

Figure 3:

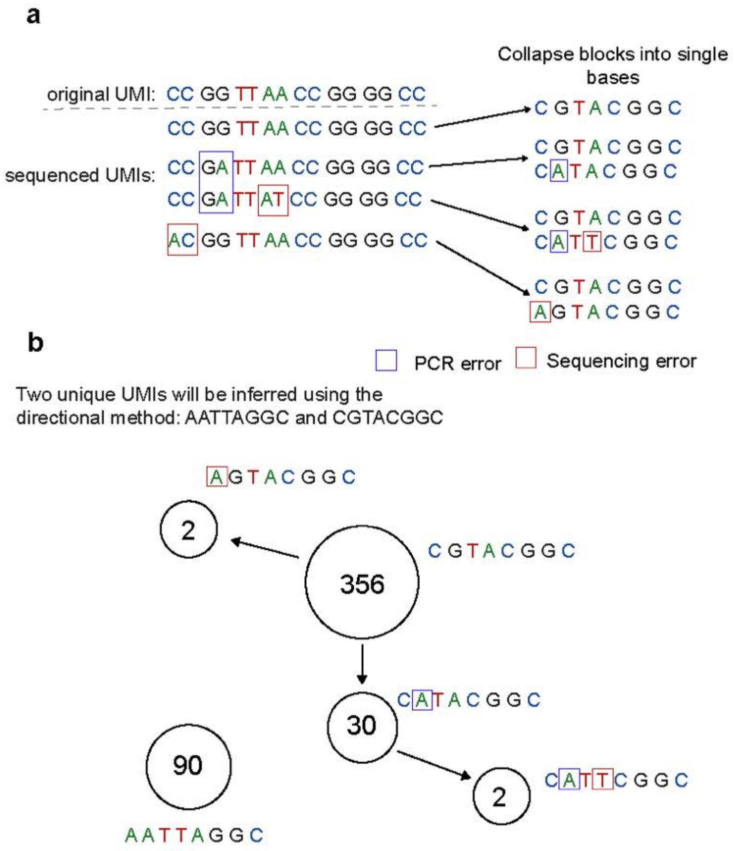

UMI counts correction strategy

We adapted the directional approach first proposed by UMI-tools for correcting PCR and sequencing errors within UMI sequences. **a**. Representation of theoretical sequencing and PCR errors within a UMI of two independent UMIs. During analysis, we first evaluate homodimer nucleotide complementarity. Molecules that show perfect homodimer complementarity within the 16mer molecule are collapsed to a single 8mer. For molecules with a mis-match in the dimer's nucleotides, these are inferred as having a sequencing error, we then split the read into two 8mer molecules. Each of these molecules are then added as independent UMIs to the UMI-tools directional algorithm (Thus an error containing molecule adds two counts to the directional algorithm). Red boxes indicate a hypothetical sequencing error and a blue box indicates a hypothetical PCR error. **b**. A diagrammatic representation of the directional network showing the two independent UMIs with sequencing and PCR errors shown in **a**. Circles with numbers represent the hypothetical counts of each sequenced molecules.

Figure 4

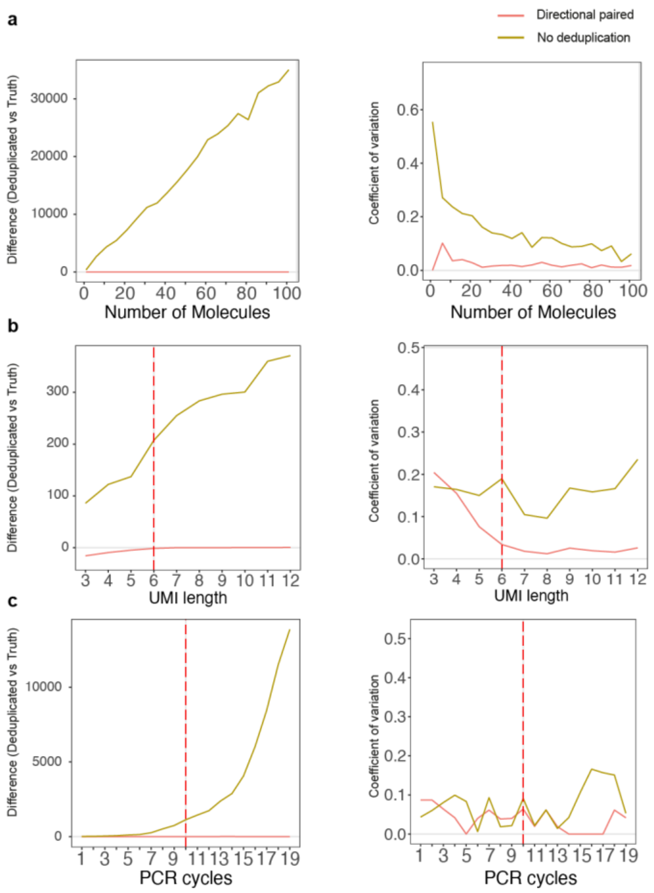

Simulated data showing the application of directional UMI deduplication strategy

Simulated data showing the difference and coefficient of variation between deduplicated UMIs and the ground truth. The left-hand pane shows the difference between the method deduplication UMI numbers and the simulated ground truth. The right-hand pane shows the coefficient of variation following 10 iterations. **a** The effect of increasing the starting number of molecules on the ability to deduplicate UMIs. **b** The effect of increasing the UMI length on the ability to deduplicate UMIs. **c** The effect of increasing the number of simulated PCR cycles using an error rate of $1x10^{-5}$.

Figure 5

Evaluating different edit distances for correcting Illumina scRNA sequencing data.

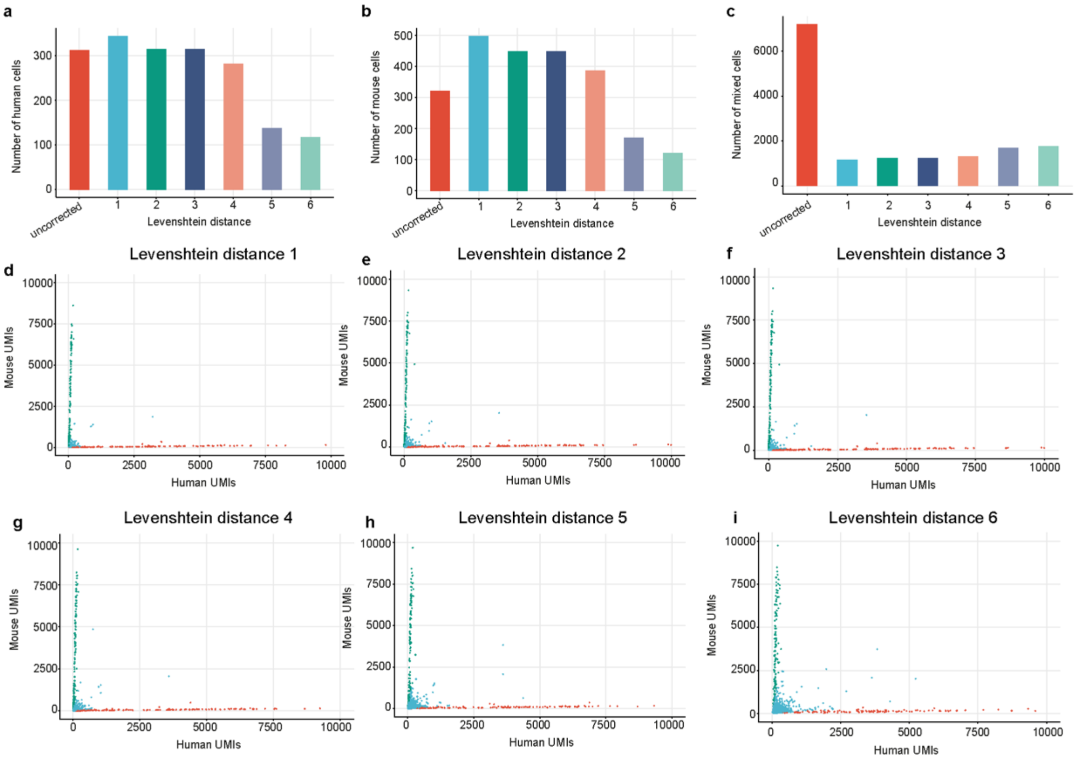

A dual oligonucleotide scRNA-seq library was generated and around 500 human HEK293T and mouse 3T3 cells were sequenced using the Illumina platform. Barcodes that contained a sequencing error, as determined by dual nucleotide block complementarity were identified. Barcodes were then error corrected using increasing edit distances. **a** The number of human cells identified using increasing Levenshtein distance for barcode error correction. **b** The corresponding numbers of mouse cells identified with increasing Levenshtein distance. **c** The corresponding numbers of mixed cells identified with increasing Levenshtein distance. **d, e, f, g, h, i** Barnyard plots showing mouse and human UMIs detected per cell.

Figure 6

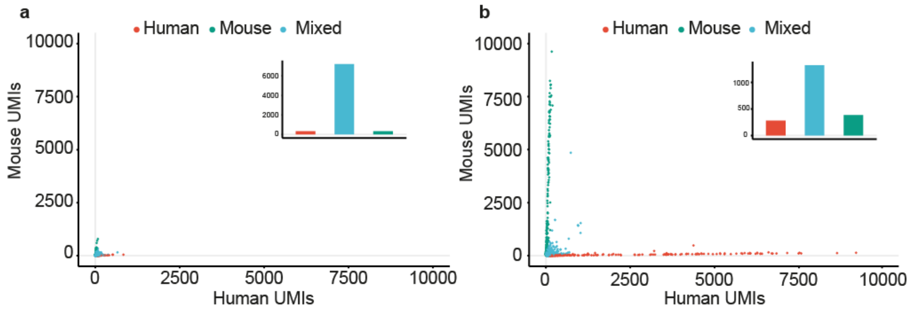

Error correction of Illumina droplet based scCOLOR-seq data.

Human HEK293T and mouse 3T3 were mixed at a 1:1 ratio and approximately 500 cells were taken for encapsulation and cDNA synthesis. Barcodes and UMIs identified as having at least one sequencing error were processed using before **a** and after barcode error correction using an edit distance of 4 **b** and the proportion of mouse and human UMIs shown in the Barnyard plot. Insert bar plots show the number of cells identified for each species.

Figure 7

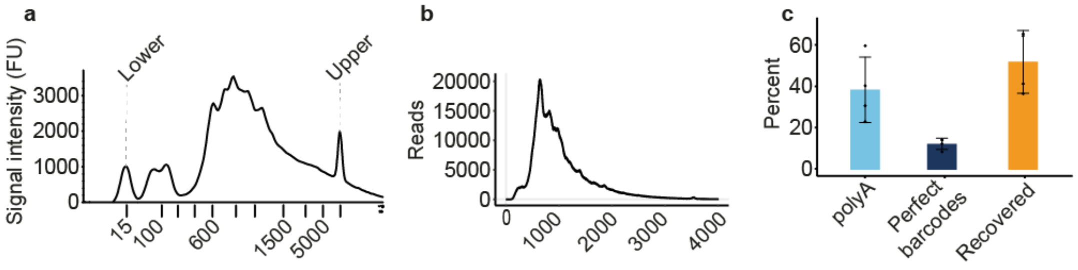

Library preparation and Nanopore sequencing outputs

a, The length of the input cDNA Nanopore library, as measured using a tapestation**. b,** The read length of the sequenced Nanopore library**. c,** The percent of reads that have a polyA tail. The percent of polyA$^+$ reads that show perfect based on the nucleotide pairing complementarity and the percent of reads that can be recovered using a Levenshtein distance of 6. Boxes and error bars indicate the means and standard deviations for n=4 individual experiments.

Figure 8

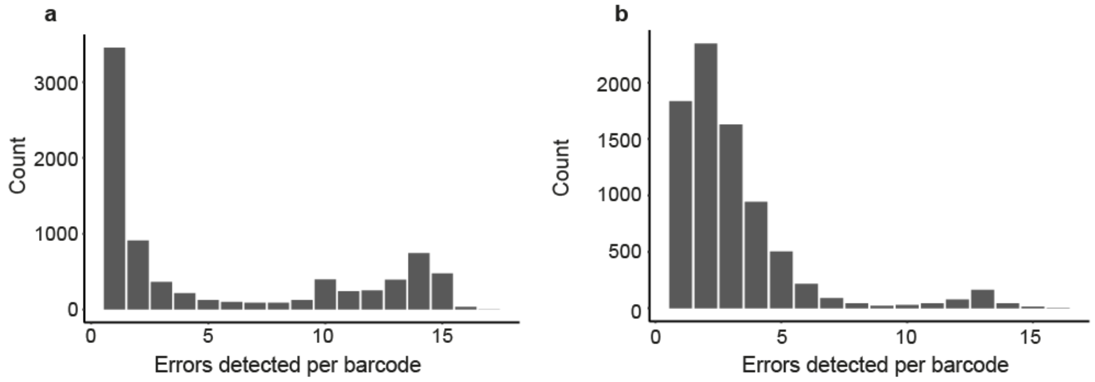

The frequency of errors within barcodes that contain at least one error.

HEK and 3T3 cells were encapsulated at a 50:50 ratio and then a library was prepared for both Illumina and Nanopore sequencing using the same cDNA**. a** 8000 Illumina sequenced barcodes were randomly selected from barcodes that contained at least one sequencing error. The frequency of sequencing errors is plotted as a bar graph. **b** Similar for **a**, but for nanopore sequenced barcodes.

Figure 9

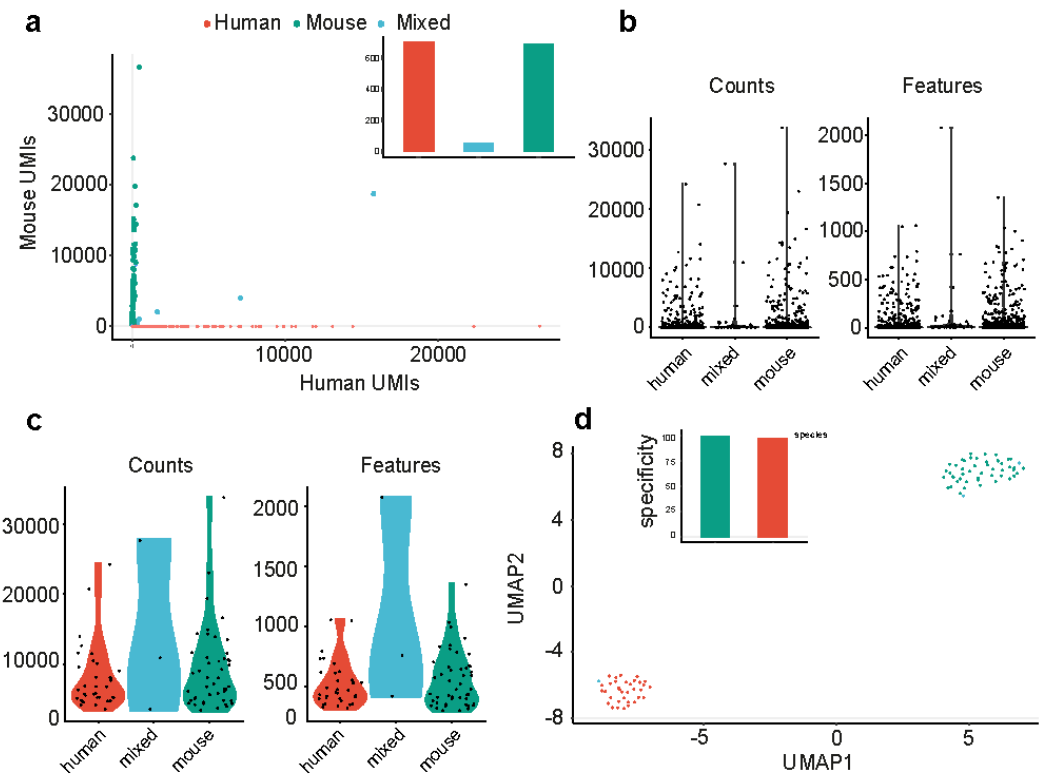

Naive collapsing of dimer bases of the barcode and UMI sequences into single bases

A companion figure for Figure 2f-I. Dimer bases within the barcode and UMI sequences were collapsed by removing the second base for each dimer pair. **a** Barnyard plot showing the expression of mouse and human UMIs prior to filtering. **b** The number of counts and number of expressed features for each cell. **c** The number of counts and number of expressed features per cell following filtering using a threshold of a minimum of 200 genes expressed per cell. **D** Post filtered cells displayed on a two-dimensional UMAP plot. The insert bar shows the specificity of UMIs aligning to either mouse or human UMAP clusters.

Figure 10

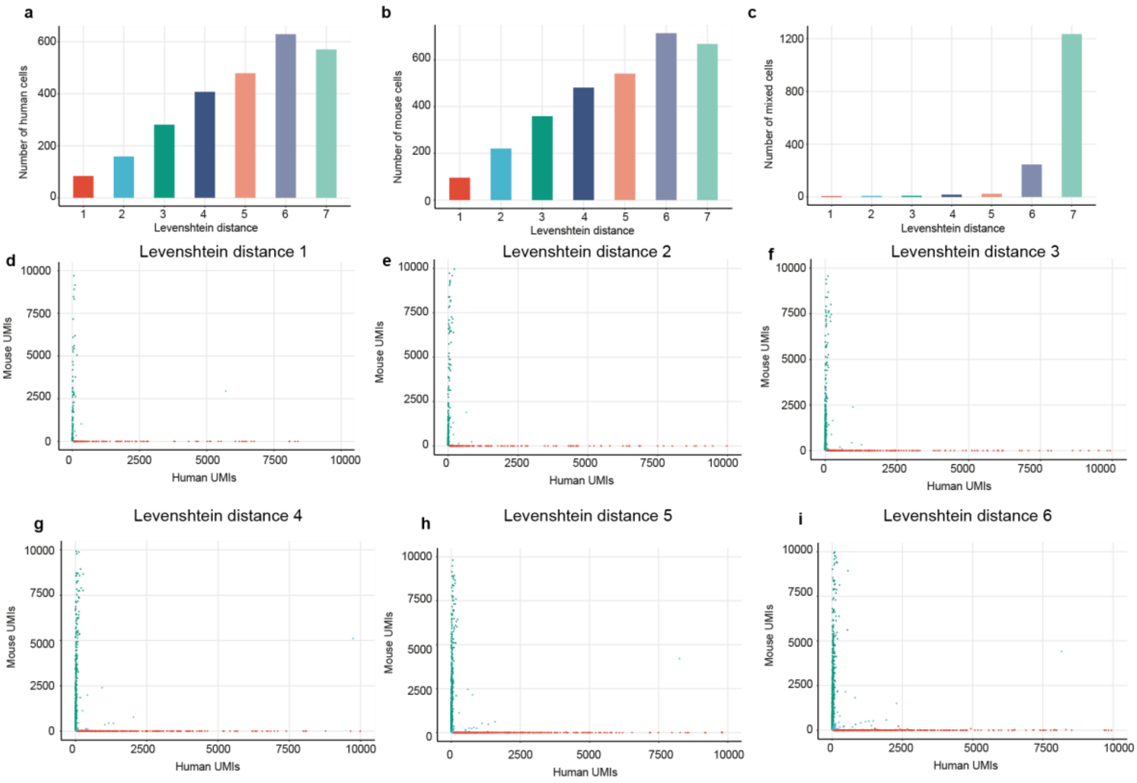

Evaluating different edit distances for correcting Nanopore scRNA sequencing data.

A dual oligonucleotide scRNA-seq library was generated and around 500 human HEK293T and mouse 3T3 cells were sequenced using the Oxford Nanopore platform. Barcodes that contained a sequencing error, as determined by dual nucleotide block complementarity were identified. Barcodes were then error corrected using increasing edit distances. **a** The number of human cells identified using increasing Levenshtein distance for barcode error correction. **b** The corresponding numbers of mouse cells identified with increasing Levenshtein distance. **c** The corresponding numbers of mixed cells identified with increasing Levenshtein distance.

d, e, f, g, h, i Barnyard plots showing mouse and human UMIs detected per cell.

Figure 11

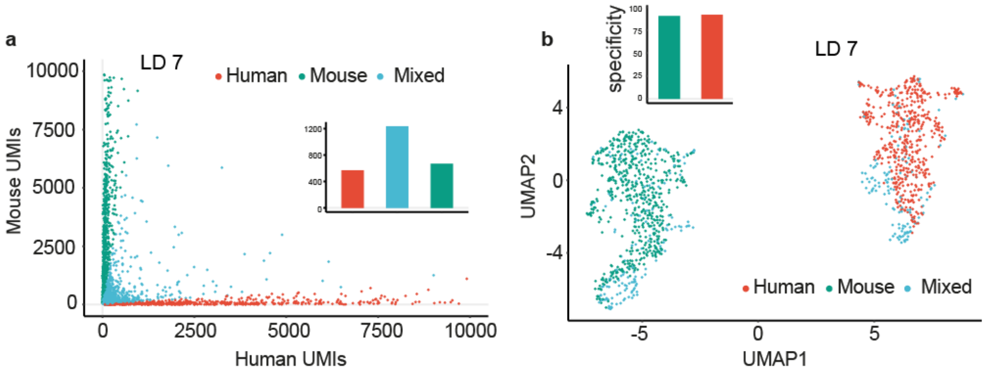

Evaluating the effect of increasing the edit distance to 7 for cell assignment.

a, A Barnyard plot showing the expression of mouse and human UMIs prior to quality filtering using a Levenshtein distance (LD) of 6. **b**, A UMAP plot of post quality filtered data showing the clustering of human, mouse or mixed human and mouse cells following barcodes correction using a Levenshtein distance of 6. The insert bar plots show the specificity of UMIs aligning to either the human or mouse UMAP cluster.

Figure 12

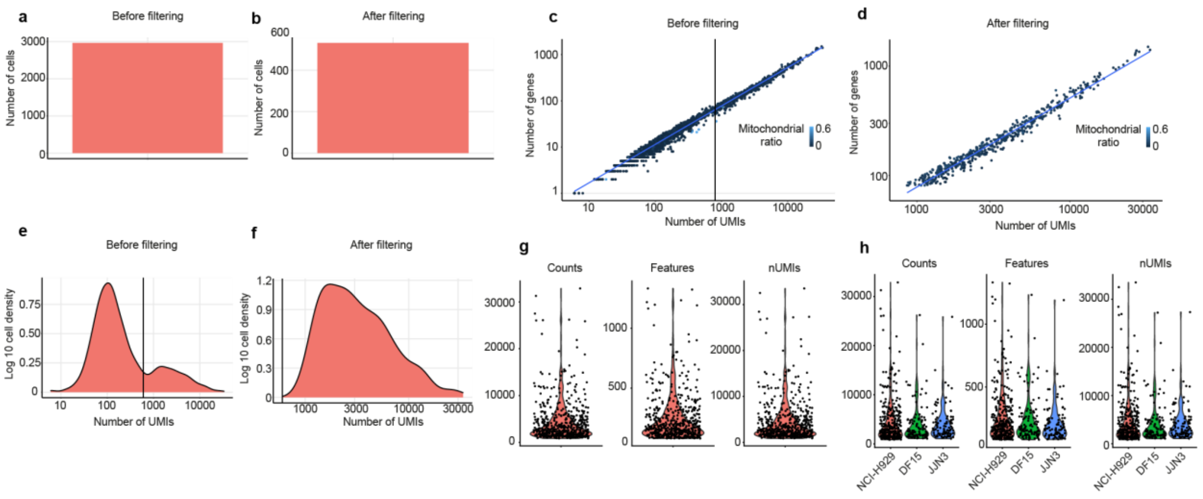

Filtering and removal of low-quality cells from 500 NCI-H929, JJN3 and DF15 mixed cell experiment sequenced using a MinION.

Cells expressing greater than 600 UMIs and 80 genes per cell was used as a threshold to filter poor quality cells from our 500-cell mixed myeloma cell line dataset. The number of cells **a** before and **b** after filtering. The relationship between the number of UMIs and the number of genes **c** before and **d** after filtering. A histogram of the number of UMIs **e** before and **f** after filtering. The number of counts, features and UMIs across **g** all filtered cells and across each **h** myeloma cell type.

Figure 13

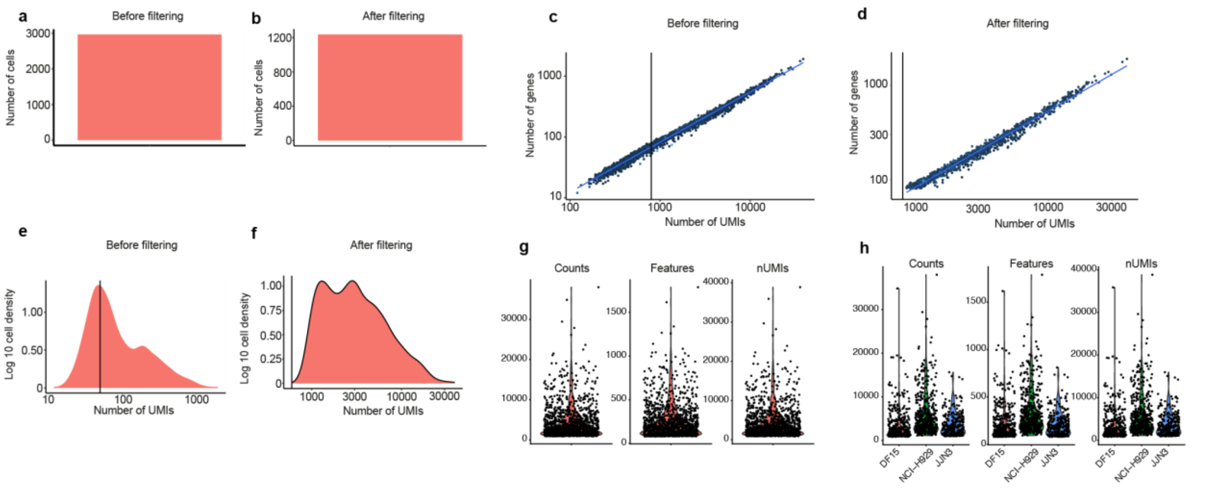

Filtering and removal of low-quality cells from 1200 NCI-H929, JJN3 and DF15 mixed cell experiment sequenced using a PromethION.

Cells expressing greater than 600 UMIs and 80 genes per cell was used as a threshold to filter poor quality cells from our 1200-cell mixed myeloma cell line dataset. The number of cells **a** before and **b** after filtering. The relationship between the number of UMIs and the number of genes **c** before and **d** after filtering. A histogram of the number of UMIs **e** before and **f** after filtering. The number of counts, features and UMIs across **g** all filtered cells and across each **h** myeloma cell type.

Figure 14

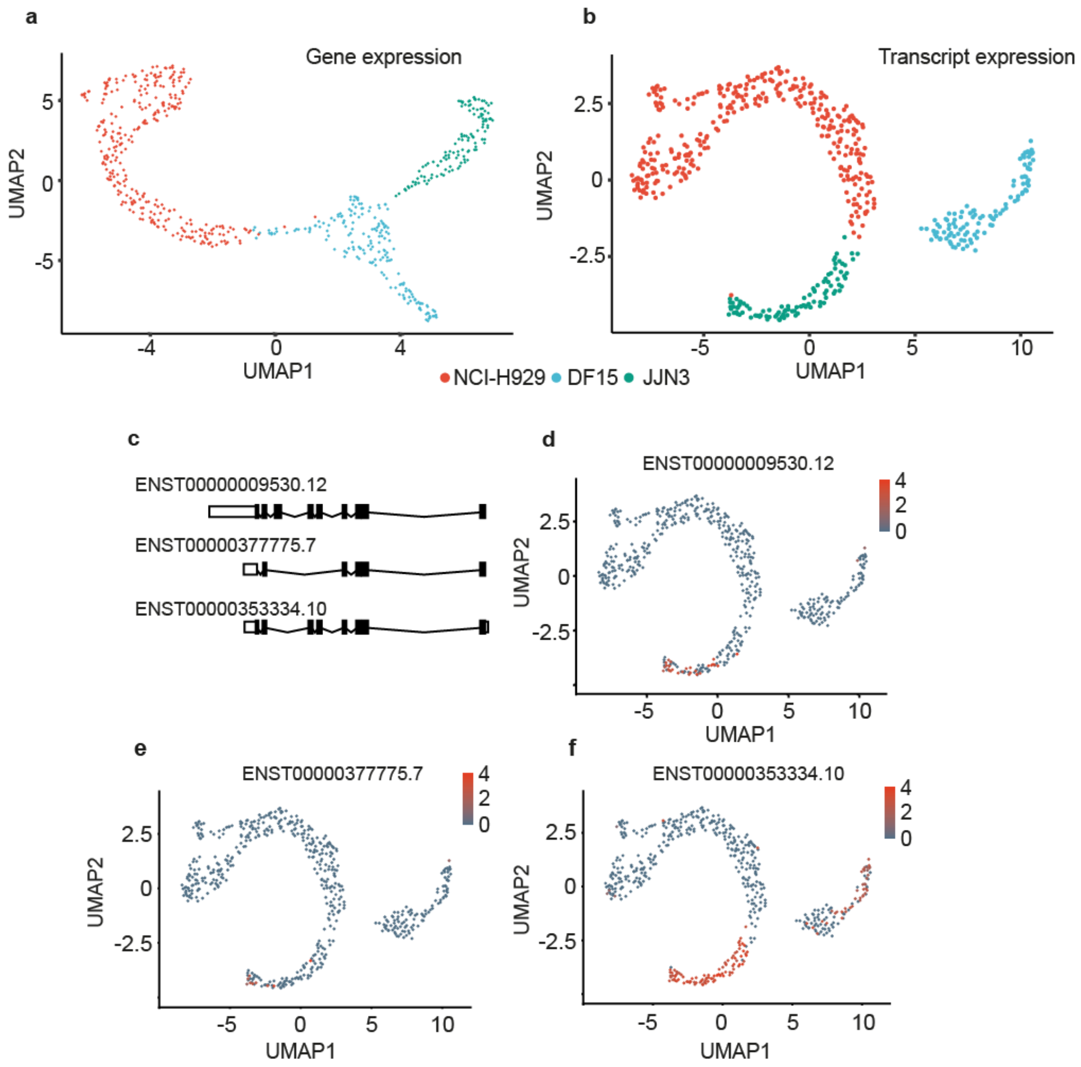

Nanopore droplet based scRNA-seq identifies isoform diversity in the 500 myeloma MinION sequenced experiment.

NCI-H929, DF15 and JJN3 myeloma cell lines were mixed at a 1:1:1 ratio and approximately 500 cells were taken for cDNA synthesis and sequenced using a MinION flow cell. UMAP plot of **a** gene expression and **b** transcript isoform expression. **c** Principal CD74 (HLA-DR) splice variants showing all protein coding transcripts. UMAP plot showing the isoform expression of detected CD74 (HLA-DR) transcripts **d** ENST00000009530.12, **e** ENST00000377775.7 and **f** ENST00000353334.10.

Figure 15

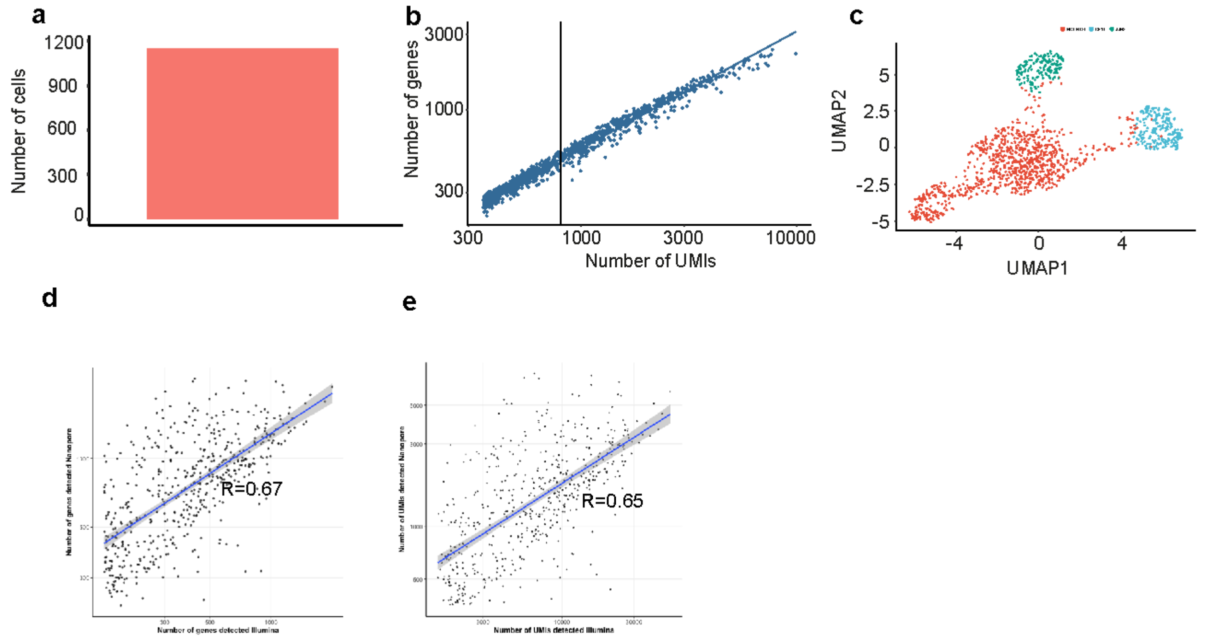

Correlation between Illumina and Nanopore sequencing

NCI-H929, DF15 and JJN3 myeloma cell lines were mixed at a 1:1:1 ratio and approximately 1200 cells were taken for cDNA synthesis (The same library that was sequenced in Figure 3) and sequenced using the Illumina platform. Cells expressing greater than 200 features per cell was used as a threshold to filter poor quality cells. **a** The number of cells after filtering. **b** The relationship between the number of UMIs and the number of genes after filtering. **c** UMAP plot of gene expression. **d** The correlation between the number of genes detected by Illumina sequencing and Nanopore sequencing. **e** The correlation between the number of UMIs detected by Illumina sequencing and Nanopore sequencing. **d**, **e** The shaded area around the line of best fit show the 95% confidence level interval predictions from the linear model.

Figure 16

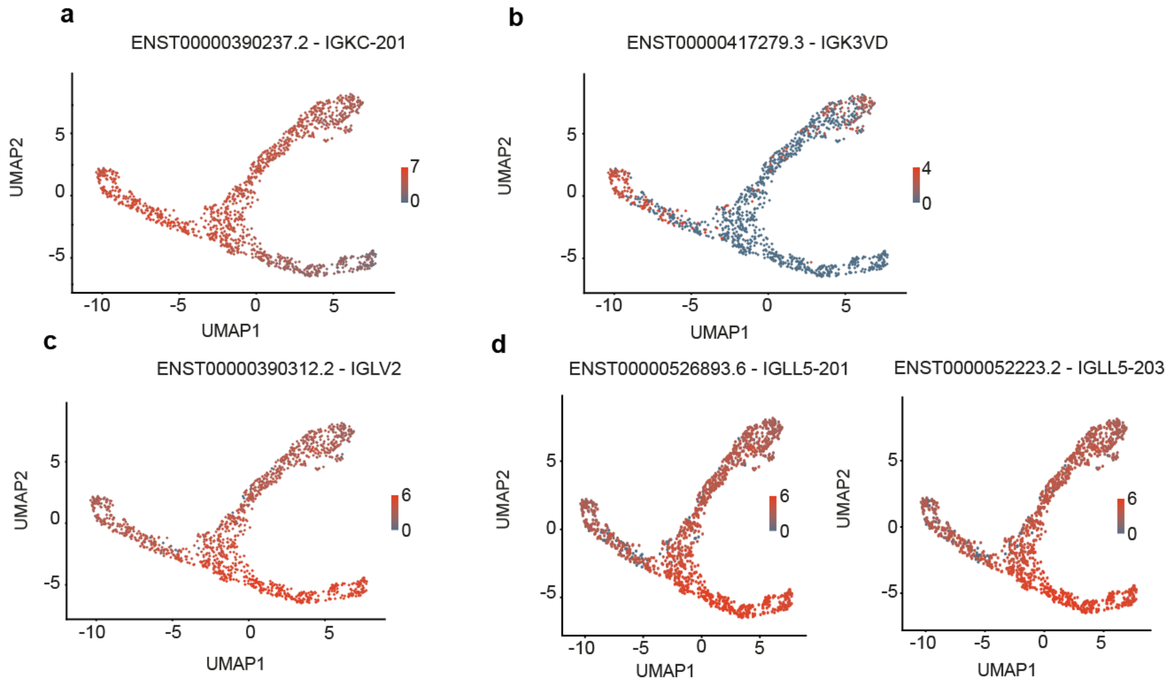

The expression of Immunoglobulin Kappa and Lambda constant transcripts in the 1200 cell myeloma experiment.

UMAP plot showing the expression of **a** IGKC-201, **b** IGKV3D-15, **c** IGLV2 and **d** IGLL5.

Figure 17

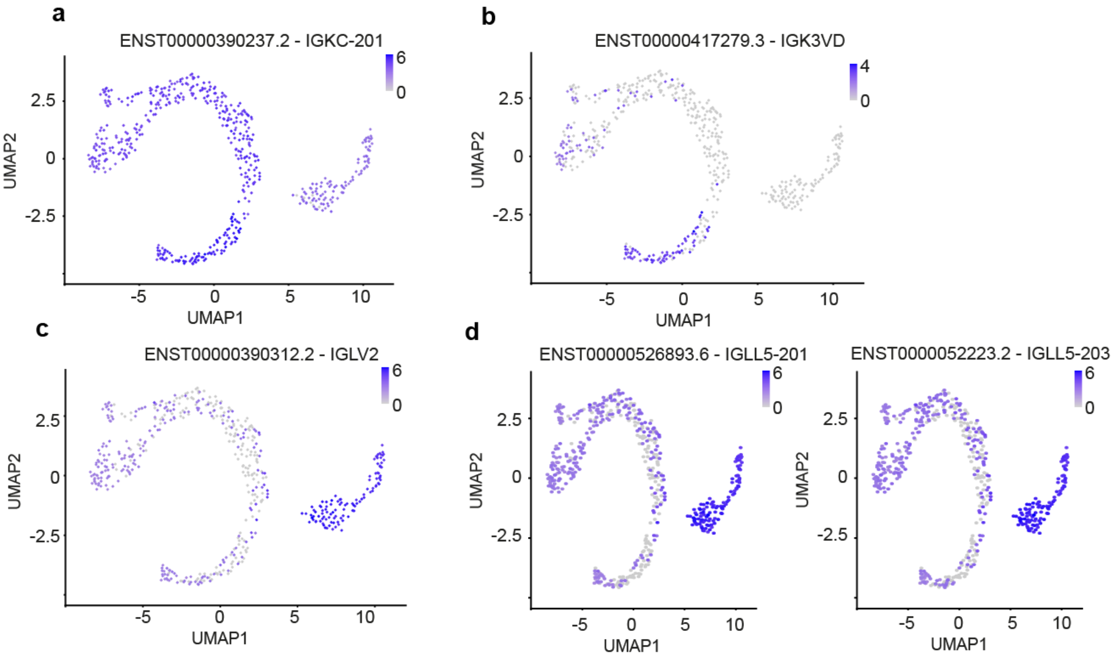

The expression of Immunoglobulin Kappa and Lambda constant transcripts in the 500 cell myeloma experiment.

UMAP plot showing the expression of **a** IGKC-201, **b** IGKV3D-15, **c** IGLV2 and **d** IGLL5.

Figure 18

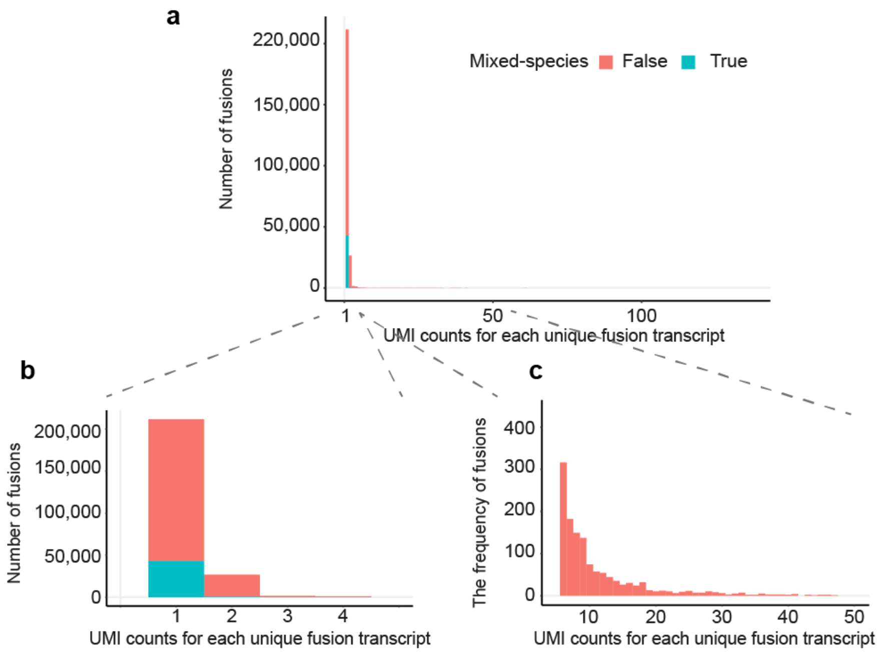

The detection of fusion transcripts within the mixed species dataset

We measured the presence of fusion transcripts within our mouse and human mixed species experiment. **a** The frequency of UMI counts per unique fusion transcript. b The same data shown in **a,** but limited to UMI counts between 1 and 5. **c** The same data shown in **a** but limited to UMI counts between 5 and 50. The colours indicate the presence of mixed species.

Figure 19

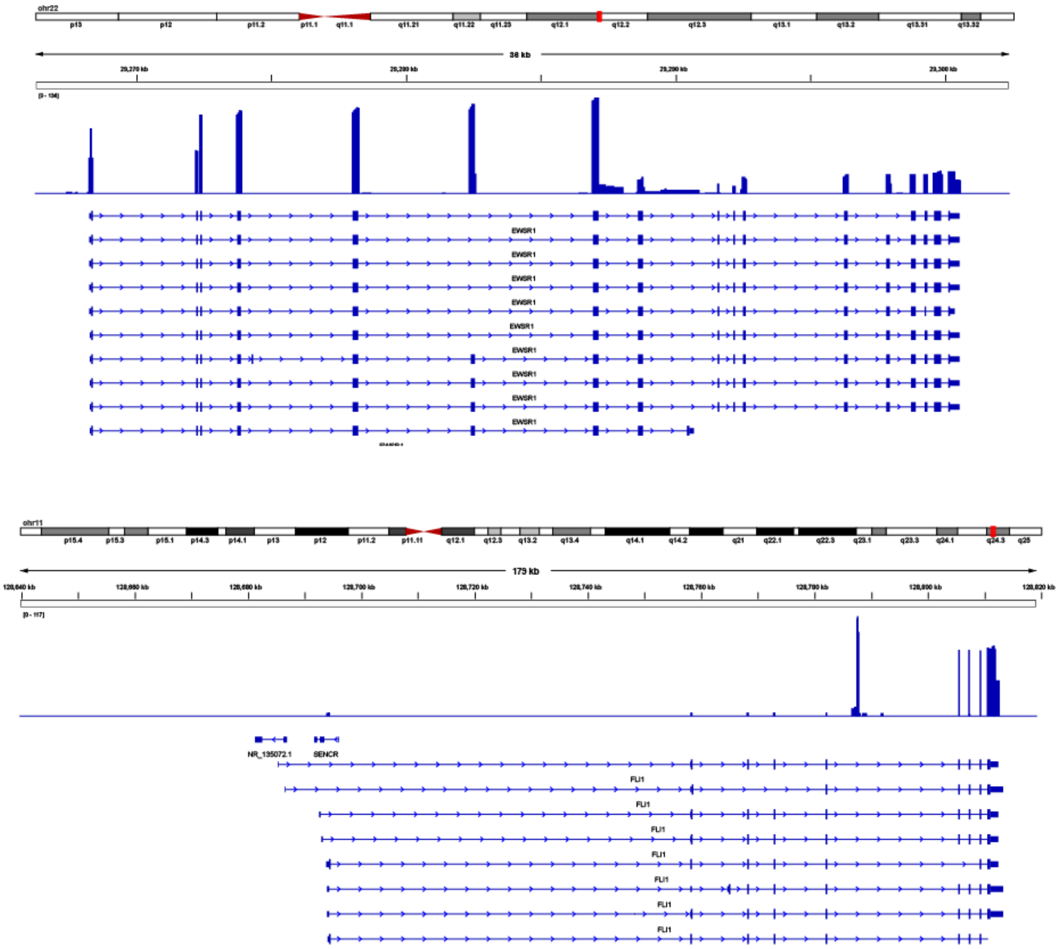

Genome browser tracks showing the read pile up across both EWSR1 and FLI1

The top panel shows the read pileup across the exons of EWSR1. The bottom panel shows the read pileup across the FLI1 gene. The peak observed within the intronic region between exons 5 and 6 appears to be an alignment artifact and does not represent a real peak.
